# Striving toward team-based continuity: provision of same-day access and continuity in academic primary care clinics

**DOI:** 10.1186/s12913-019-3943-2

**Published:** 2019-03-04

**Authors:** Jane H. Forman, Claire H. Robinson, Sarah L. Krein

**Affiliations:** 1 VA Center for Clinical Management Research, VA Ann Arbor Healthcare System, UM North Campus Research Complex, 2800 Plymouth Road, Building 16, 3rd floor, Ann Arbor, MI 48109-2800 USA; 2 Department of Internal Medicine, University of Michigan, Ann Arbor, MI USA

**Keywords:** Primary care redesign, Continuity of care, Access to care, Medical education, Qualitative research

## Abstract

**Background:**

An important goal of the patient-centered medical home is increasing timely access for urgent needs, while maintaining continuity. In academic primary care clinics, meeting this goal, along with training medical residents and associated professionals, is challenging.

**Methods:**

The aim of this study was to understand how academic primary care clinics provide continuity to patients requesting same-day access and identify factors that may affect site-level success. We conducted qualitative interviews from December 2013–October 2014 with primary care leadership involved with residency programs at 19 Veterans Health Administration academically-affiliated medical centers. Interview recordings were transcribed verbatim. To analyze the data, we created comprehensive, structured transcript summaries for each site. Site summaries were then entered into NVivo 10 software and coded by main categories to facilitate within-case and cross-case analyses. Themes and patterns across sites were identified using matrix analysis.

**Results:**

Interviewees found it challenging to provide continuity for same-day in-person visits. Most sites took a team-based approach to ensure continuity and provide coverage for same-day access, notably using NPs, PAs, and RNs in their coverage algorithms. Further, they reported several adaptations that increased multiple types of continuity for walk-in patients, urgent care between in-person visits, and follow-up care. While this study focused on longitudinal continuity, both by individual PCPs or by a team of professionals, informational continuity and continuity of supervision, as well as, to a lesser extent, relational and management continuity, were also addressed in our interviews. Finally, most interviewees reported clinic intention to provide patient-centered, team-based care and a robust educational experience for trainees, and endeavored to structure their clinics in ways that align these two missions.

**Conclusions:**

In contending with the tension between providing continuity and educating new clinicians, clinics have re-conceptualized continuity as team-based, creating alternative strategies to same-day visits with a usual provider, coupled with communication strategies. Understanding the effect of these strategies on different types of continuity as well as patient experience and outcomes are key next steps in the further development and dissemination of effective models for improving continuity and the transition to team-based care in the academic clinic setting.

**Electronic supplementary material:**

The online version of this article (10.1186/s12913-019-3943-2) contains supplementary material, which is available to authorized users.

## Background

An important goal of the patient-centered medical home (PCMH) is increasing timely access for urgent needs, while maintaining continuity [1, 2]. Indeed, the National Committee for Qualitative Assurance (NCQA) 2017 PCMH Recognition standards include same-day access and provision of continuity using team-based approaches. Meeting these standards in academic primary care clinics, where multiple part-time physicians, and associated professionals, provide care, may be particularly challenging [3–5]. Many primary care physicians (PCPs) and residents in academic clinics are only available only for clinical care part-time, as they participate in training, research, administrative, or other clinical activities [6]. Consequently, the risk of discontinuity is higher for patients who request same-day access compared to routine visits [7]. Understanding how academic clinics structure delivery of such care is important to mitigate potential negative consequences [8].

In addition to care delivery, academic clinics play a critical role in training medical residents and other clinical professionals in team-based care. The National Academy of Medicine defines team-based care as “...the provision of health services to individuals, families, and/or their communities by at least two health providers who work collaboratively with patients and their caregivers—to the extent preferred by each patient - to accomplish shared goals within and across settings to achieve coordinated, high-quality care” [9]. In the PCMH model, the PCP shares primary care of a defined patient panel with team members such as registered nurses (RNs), licensed practical nurses (LPNs), pharmacists, and administrative staff, ideally with well-defined team roles and processes. Training in team-based care is essential since “sharing the care” among team members [10] is fundamental to improving access and providing more efficient and cost-effective care [11, 12].

The emphasis on team-based care also signals a shift in the traditional conceptualization of continuity as consisting of in-person visits over time with a usual PCP, usually termed longitudinal continuity [13, 14]. Longitudinal continuity is a dimension of relational continuity that is necessary for the development of a therapeutic relationship. Although there are strategies to balance individual provider and team-based longitudinal continuity [15] (i.e. seeing the same team of professionals over time), team-based care in the training environment, with multiple PCPs and residents on a team, can pose additional challenges. And, because longitudinal continuity is more likely to be disrupted in a training environment, other types of continuity -- management continuity (i.e., consistency of clinical management) and informational continuity (i.e., use of information on past events and patient characteristics to deliver appropriate care to a particular patient) [13] – are also more challenging than in an environment with full time PCPs. Structures and processes must be put in place to share information among multiple PCPs, in addition to other primary care team members. In the academic setting, an additional type of continuity to consider is continuity of supervision, that is, a resident having the same attending supervisor over time in caring for the resident’s patient panel [16]. This provides the potential benefits of longitudinal continuity to both residents and patients.

In 2010, the Veterans Health Administration (VHA) began transitioning its primary care clinics to the PCMH model [17–19]. Approximately 120 of the over 900 VHA primary care clinics are academically affiliated and located within medical centers. These clinics fulfill VHA’s dual mission of patient care and medical training. VHA funds about 15% of US internal medicine resident positions, and has instituted innovative inter-professional education that trains medical residents and Nurse Practitioners (NPs) together as part of care teams [20]. This transformation provided the ideal opportunity to understand potential challenges and practical strategies for achieving access and continuity goals during the transition to team-based care within the academic primary care clinic training environment. The focus of this project was on longitudinal continuity, but we also report findings on management and informational continuity, and continuity of supervision, as they arose in our interviews. Specifically, we conducted a multi-site qualitative assessment to: 1) Understand how academic primary care clinics approach providing continuity for patients requesting same-day access; 2) Identify factors that affect site-level success in providing such continuity; and 3) Describe adaptations that clinics have developed to provide continuity. We chose this approach to gain an in-depth understanding of the views and experiences of clinic leaders in dealing with same-day access and continuity issues.

## Methods

### Setting

This multi-site quality improvement project took place 3 years after the VHA implemented PACT (Patient Aligned Care Teams), VHA’s version of PCMH, in which care is provided through an ongoing relationship with a designated team of clinical and administrative staff. During this time, one area of focus was same-day access, promoted by a performance measure that assessed the proportion of patient requests for same-day appointments that resulted in a same-day in-person appointment with the patient’s usual PCP (longitudinal continuity, hereafter continuity). For residents, performance was measured at the level of the supervising attending physician. This reinforced the benefit of having the same attending involved with a patient’s care, as well as continuity of supervision. If an attending had their own patient panel, performance for that panel was measured separately. All VHA primary care clinics are internal medicine clinics.

### Sampling and data collection

We sent recruitment e-mails to physician clinic administrators involved with the residency program and primary care clinic management at 1 academic medical center in each of the 21 regional Veterans Integrated Service Networks (VISNs), to ensure geographic variation. We aimed to recruit physician administrators because they were most familiar with the residency program; when that person was unavailable, we interviewed another administrator. We were unable to arrange interviews in 5 VISNs, so selected 3 additional medical centers from 3 of the already-participating VISNs to supplement the study sample. Two authors (JF and CR) developed an interview guide with input from the larger study team (Additional file 1). The guide was designed to collect data on a common set of domains across interviewees based on our research questions and our earlier work on PCMH implementation [6]. Domains included clinic structure, resident model, coverage for residents absent from clinic, part-time PCPs, and main challenges. JF and CR conducted semi-structured telephone interviews with 17 physicians and 2 nurse practitioners. Both interviewers participated in the interviews, which occurred from December 2013 through October 2014 and lasted approximately 1 h. For the 2 interviewees who did not grant permission to audio-record the interviews, we captured interview content with thorough notes. Recorded interviews were transcribed verbatim [21].

### Data analysis

From the transcripts and notes, we created comprehensive structured summaries. First, we created a template for the summaries based on immersion in the data, including post-interview discussions, with the goal of addressing the research questions. The template was further refined by constructing summaries for 2 sites and subsequently used to create summaries for all sites. For each category, summaries included descriptive and interpretive comments, as well as supporting exemplar quotations that comprehensively represented the data. Site summaries were then entered into NVivo 10 software and coded by main categories to facilitate within-case and cross-case analyses. Specifically, we created a descriptive matrix with defined rows (one row for each site) and columns (main categories) so we could view and compare summary site characteristics, processes, and recommendations [22]. We identified themes by reviewing data in each category and identifying connections between categories and patterns across sites. To ensure interpretive rigor, we went back to original transcripts when needed to verify interpretations and conclusions [23]. Rigor was also strengthened through the detailed nature of the interviews and discussion of findings with members of a wider team involved in evaluating the PCMH model in VHA, including local primary care administrators and PCPs.

## Results

The characteristics of the 19 participating clinics varied across several dimensions, including the number of enrolled primary care patients (approximately 9000 to 100,000), the number of residents (16–144), and the number of attending physicians (5–40) (Table 1). Similarly, clinics had varied organizational structures, such as whether residents were integrated throughout the clinic or assigned to a resident-only team, the percentage of patients assigned a resident PCP (2–43%), and type of residency model (traditional vs. block). In the traditional model, residents’ schedules are dominated by inpatient care, with only a half-day per week devoted to ambulatory care. Block models alternate blocks of inpatient rotations with dedicated ambulatory blocks (e.g., 6 weeks inpatient, 2 weeks outpatient).

**Table 1:** Characteristics of participating primary care clinics (*n* = 19 in 16 VISNs)

Clinic Size	
# Primary Care patients enrolled	~ 9000 to ~ 100,000
% Primary Care patients with a resident as their assigned PCP	2 to 43%
# Residents	16^a^ to 144
# Attending physicians (i.e., precept residents)	5 to 40
Clinic Structure	
# Sites with attendings who were:	
Most or all part-time in clinic with own patient panels	15 (79%)
All full-time in clinic with own patient panels	3 (16%)
All full-time in clinic without own patient panels	1 (5%)
# Sites with residents who were:	
Integrated into most or all clinic teams	10 (53%)
On teams separate from non-academic teams, co-located with non-academic teams	7 (37%)
On teams separate from non-academic teams, not co-located with non-academic teams	2 (10%)
Residency Model	
Traditional model (1 to 2 half days in clinic per week)	13^b^ (68%)
Block model (weeks of inpatient and outpatient rotations, including 4 + 1, 4 + 4, 6 + 2, 8 + 8, and 12 + 12)	6 (32%)

*VISN* Veterans Integrated Service Network, *PCP* Primary Care Provider

^a^Site in its first year hosting a residency program

^b^8 of these were moving to or considering moving to a block model

### Approaches to providing continuity: coverage for residents absent from clinic

Most sites were trying to structure their clinics in ways that align patient care with their teaching mission. Almost all sites, however, were challenged in providing continuity to patients assigned to residents:

“one of the goals of the VA, which we know in the mission statement, is care of the Veteran…but also education...we know that trainee providers just by their very nature are not going to be in the ambulatory setting [everyday], so there’s going to be some discontinuity.” (Site P)

Processes established to provide care when residents were not in clinic were central to attempts to provide continuity to patients requesting same-day visits. Given the scheduling complexities introduced by having residents (and, at most clinics, part-time attendings), and by the focus on team-based care, 12 of 18 sites where residents had their own patient panels took a team-based approach to providing coverage for same-day in-person access (Table 2). That is, when a patient of a resident who wasn’t in clinic needed a same-day in-person visit, most sites attempted to provide team-based continuity to that patient by having someone else on the absent resident’s team -- another resident, attending physician, registered nurse (RN), NP, or physician assistant (PA) -- see the patient. Interviewees perceived maintaining team-based continuity to benefit both training and patient care (Table 3, Quote 1). There was no discernable pattern across sites of coverage arrangements varying by residency model, resident integration into clinic, or number of residents in clinic.

**Table 2:** Same-day access coverage arrangements for patients of a resident not in clinic

Site	1st choice	2nd choice	3rd choice
F	**Resident’s attending (RN if MD not needed)**	n/a	n/a
L	**Resident’s attending**	**Attending’s PA or resident**	n/a
P	**Team resident**	**Team NP Trainee**	**Team NP**
G	**Team resident**	**Team attending**	Any open slot
H	**Team resident**	**Team attending**	Any open slot
M	**Team PA**	**Team resident**	Any open slot
Q	**Team attending (RN if MD not needed)**	**Resident of team attending**	Any open slot
N	**Team resident**	Any resident	Any NP or attending
D	**Team resident**	Any resident	n/a
A	**Team attending or resident**	Walk-in clinic resident on ambulatory care rotation	n/a
S	**Team attending**	Walk-in clinic resident on ambulatory care rotation	**Team resident**
C	Any resident	**Team attending**	Any open slot
O	Any resident	Any attending	n/a
J	Any resident	n/a	n/a
I	Walk-in clinic residents on ambulatory care rotation	n/a	n/a
B	Walk-in clinic residents on ambulatory care rotation or RN	Any PCP	n/a
E	Walk-in clinic residents on ambulatory care rotation	n/a	n/a
K	Walk-in clinic residents on ambulatory care rotation	n/a	n/a
R^a^	n/a	n/a	n/a

**Bold** = within team

*RN* Registered Nurse, *MD* Physician, *PA* Physician’s Assistant, *NP* Nurse Practitioner, *PCP* Primary Care Provider

^a^Coverage arrangements for Site R’s residents are not applicable because residents did not have their own panels

**Table 3:** Supporting quotes

Quote number	Category	Quote
Approaches to Providing Continuity: Coverage for Residents Absent from Clinic		
1	Team-based continuity beneficial for both training and patient care	“Workarounds…for [urgent] access…just…adding…another resident to see patients…is not really patient-centric [and] it doesn’t engage the house staff in following those patients.” (Site A)
2	NPs, PAs, NP trainees, or RNs, not just medical residents and attendings, were included in coverage algorithms.	“…each one of our PA’s is assigned to one of the…PACT teams…the residents are all spread out among the teams…when…possible [residents] try to stick with the same PACT team for continuity” (Site M)
3	About half of participating clinics expected residents to address urgent between-visit needs.	“…we want to…encourage [residents] and help them to have a lot of ownership of these patients…there’s an expectation that they communicate with their LPN and their RN, the LPNs do a really nice job of paging them or texting them if a patient…needs to be taken care of promptly…” (Site S)
Barriers to Providing Team-based Continuity		
4	*Residency Program-related:* Low predictability of resident schedules.	“P: …when the resident’s not in clinic, they see their co-team resident and if no one on their co-team is available, then we put them in with any resident. I: How often do you think a co-team resident is there? P: It’s just hit or miss because it’s so up in the air” (Site D)
5	*Residency Program-related:* Low predictability of resident schedules.	“especially with the residency requirement and…the demand on the residents from the inpatient setting, it’s been very, very challenging to build any kind of cohesive system of continuity…care, and consistency of having the…resident in clinic.” (Site C)
6	*Residency Program-related:* Block model may increase schedule predictability	“sites that have [gone to the block model]…in terms of…predictability of schedules, access…people know when residents are going to be there and not be there… the residents themselves are also much happier.” (Site C)
7	*Clinic-related:* Attendings with duties that took them away from clinic.	“…they’re doing teaching at the medical school…running the educational portion of the resident inpatient rotation…homeless shelter work…they’re more complicated in terms of the scheduling and making sure we have adequate coverage…” (Site O)
8	Teaching continuity beneficial to training and patient care	“Having that attending-resident continuity is very helpful in teaching...we can actually teach the residents how to improve as doctors…that helps overall patient care always.” (Site D)
9	*Clinic-related:* Access pressure that limited attendings’ availability to see their residents’ walk-in patients.	“P: 10 attendings that…have interns and residents…are all in clinic today, no intern or resident is in clinic today. I: If a resident’s patient comes in on a day where that resident’s attending is there, is there any effort [made for the patient to see the resident's attending]? P: If the attending has access, but 10 times out of 10 that attending doesn’t have access” (Site B)
Adaptations to Increase Continuity		
10	NP trainees, along with two resident partners, cross-cover each other.	“…if [resident] X is not here, then his physician partner will be with his patient. If that physician partner was not here, the nurse practitioner partner would see the patient. If that person is not available, then the back-up nurse practitioner would see the patient” (Site P)
11	Huddles increase informational continuity among team members.	“…trainees feel like when they have the complex patients who are coming in frequently, you’d much rather them come to a partner than a random person who doesn’t know their story… then we could talk about it in the huddle and make a plan and all be on the same page” (Site P)
12	Residents share a patient panel	“… [we could] have 6 or 8 residents that…[have] a relatively full-time presence in the clinic…and there’s continuity and they cross-cover… they team up to provide this really nice patient-centered level of care…they learn how to collaborate and they depend on each other…” (Site C)
13	RNs available for follow-up after huddling with team.	“…that’s the whole benefit of having pre-clinic huddles…there is a lot of communication…especially between the residents and their RN care managers. The RN care manager takes care of a lot of issues in between visits.” (Site A)

Notably, 7 sites included NPs, PAs, NP trainees, or RNs, not just medical residents and attendings, in their coverage algorithm. For example, at Site M, each team had an assigned PA who was often first in line to see a walk-in patient of an absent resident on their team. At this site, residents were integrated across teams (Quote 2).

Six sites used walk-in clinics, 4 staffed by residents to see patients requiring same-day access, at 2 others, walk-in clinics were second in the coverage algorithm. Five of these 6 sites stated that residents staffing walk-in clinics were “making up” ambulatory hours mandated by the Accreditation Council for Graduate Medical Education (ACGME) that they didn’t fulfill with continuity visits with their own patients. Walk-in clinics were perceived to compromise continuity, but at all but 1 of the sites using walk-in clinics, interviewees described attempting to  provide continuity either at the attending physician level or by the team RN.

About half of the sites expected residents to address urgent between-visit needs, through checking and responding to electronic health record view alerts and, at some sites, being available to their team via page, text, or secure e-mail (Quote 3), with attending physicians or RNs responsible for assuring timely patient care.

### Barriers to providing team-based and other types of continuity

Interviewees described the following barriers to providing continuity for both same-day and routine visits.

#### Residency program-related barriers

Low predictability of resident clinic schedules was thought to make it more challenging for a co-team resident to see an absent resident’s walk-in patient (Quote 4), affecting team-based continuity for same-day visits. It was also more difficult to have residents and their assigned attendings in clinic at the same time, affecting continuity of supervision and team attending-level continuity for both same-day and routine visits. About half of interviewees described their academic affiliate as putting constraints on residents’ schedules. (Quote 5). For example, at Site D, with a traditional residency model, residents often changed their clinic date because they were post-call (i.e., just finished an overnight inpatient shift) or “on a rotation they can’t get out of.” This seemed to primarily affect continuity for routine visits for patients with a resident PCP.

Three facilitators of higher resident schedule predictability, and therefore potentially continuity, included: 1) a move toward the block model (Quote 6); 2) the ability of leadership to negotiate scheduling with the academic affiliate; and 3) early communication with their academic affiliate regarding scheduling.

#### Clinic-related barriers

The first clinic-related barrier to providing continuity was lack of attending physician availability. Fifteen sites had attending physicians with duties other than precepting residents and seeing their own patients. These duties took them away from clinic, and made it difficult to see patients of residents they supervised, or patients of other residents on their team (Quote 7). One site had attending physicians who were in clinic full-time either precepting or seeing their own patients, but because they were under pressure to provide access, they were too busy to see their residents’ walk-in patients (“I don’t see how you would ever get anything done.” Site K).

Second, clinics with a large number of residents and/or attending physicians found it difficult for attending physicians and their residents to be in clinic at the same time to provide attending--level continuity to patients and continuity of supervision to residents. And, even high resident schedule predictability did not guarantee high continuity of supervision. At Site J, with 18 part-time attendings who were in clinic infrequently, team RNs provided continuity instead of attending physicians. Site N had both multiple part-time attending physicians *and* low resident schedule predictability, and, to facilitate team-based continuity, NPs “provided the glue.” Interviewees saw continuity of supervision as beneficial for both training and patient care (Table 3, Quote 8).

The third clinic-related barrier was that multiple schedulers lacked knowledge of coverage arrangements, and had difficulty dealing with scheduling for a large number of residents. These barriers affected sites’ ability to match patients with their assigned provider (e.g., Site M, with 70 residents). Site P, with 50 residents, reported addressing this problem by programming their coverage algorithm in the electronic medical record that all schedulers could access.

Finally, scarcity of open visit slots for walk-in patients made it more difficult for attending physicians to see their residents’ walk-in patients. Site B reported that attending physicians, who were all part-time, rarely had open slots, and had a difficult time providing coverage for their assigned residents (Quote 9).

### Adaptations to increase team-based continuity

Sites described several adaptations to increase team-based continuity for walk-in patients, as well as for routine care, urgent care between in-person visits, and follow-up care. Adaptations included expanding the types of team members in the coverage algorithm, using team full-time attending physicians or co-team residents, and using other modalities for providing care.

For example, a team NP, PA, or RN provided coverage for walk-in patients at several sites (Table 2). One site noted that including within-team RN visits would provide another layer of continuity, as the team RN would see the patient instead of a non-team provider. Site P included NP trainees to form a “practice partner triad”, consisting of 2 resident partners and an NP trainee who each had their own panels, but cross-covered one another. This site reported being quite successful at providing team-based continuity (Quote 10); team meetings (i.e., huddles) strengthened informational continuity through discussion of care of individual patients (Quote 11).

Site L depended on attendings who were full-time preceptors to provide continuity for their residents’ walk-in patients: “Same day access is not a problem really…the attending will see the patient.” Site S increased the number of attending physicians who were full-time preceptors to improve continuity. On the other hand, Site G paired co-team residents so they had the same patient panel, and could address between-visit needs while their partner was on inpatient rotation. Site A had a co-team resident who was in clinic the subsequent week to manage test results. Two interviewees commented that resident cross coverage and sharing of patient panels was positive for training and patient care. (Quote 12).

In keeping with the PCMH model, sites also urged residents to use secure e-mail and telephone appointments for care between clinic days, thus strengthening relational continuity. Another strategy, having RNs available for follow-up after huddling with residents about the care of individual patients, was aimed at using informational continuity to improve patient management. (Quote 13).

## Discussion

This is the first multi-site, qualitative, in-depth look at how academic primary care clinics transitioning to the PCMH model approach providing continuity for patients requesting same-day access. Most sites took a team-based approach to ensure continuity and provide coverage for same-day access, notably using NPs, PAs, and RNs in their coverage algorithms. Further, they reported several adaptations that increased multiple types of continuity for walk-in patients, urgent care between in-person visits, and follow-up care. While this study focused on longitudinal continuity, both by individual PCPs or by a team of professionals, informational continuity and continuity of supervision, as well as, to a lesser extent, relational and management continuity, were also addressed in our interviews. Finally, most interviewees reported clinic intention to provide patient-centered, team-based care and a robust educational experience for trainees, and endeavored to structure their clinics in ways that align these two missions.

We found that coverage arrangements that promoted team-based care and continuity could be at the PCP level (i.e., resident and resident’s attending, team resident, NP, or PA) or with the wider team (i.e., RN). These strategies are like those reported as improving access and continuity by a public hospital resident primary care clinic [24]. When thinking about improving clinic processes, it is important to note that processes to ensure informational and management continuity, in addition to longitudinal continuity, would depend on which team member is providing coverage. For example, a resident and the resident’s attending have regular opportunities to exchange patient information and to think about management of the patient as part of the resident’s supervision by the attending. On the other hand, natural opportunities for a resident and an RN to exchange information may be more limited, and processes such as clear and accessible documentation in the electronic medical record, or huddles would be needed to bridge that gap.

Team-based coverage arrangements can have little positive effect on patient care without informational continuity. Strategies for achieving informational continuity are important to team-based care generally, and especially in academic clinics that have multiple part-time PCPs on a team. Some interviewees reported that intra-team communication through huddles and electronic means facilitated communication. These strategies have potential to provide the informational continuity essential to filling “continuity gaps” in the context of part-time providers and team-based care [3, 25–28]. Brown et al. found that an increase in informational continuity in an academic PCMH clinic through information technology and team meetings improved management continuity across the team, including for urgent patients not seeing their assigned provider [26]. NCQA recognized the importance of informational continuity by adding a new element to its 2014 PCMH certification standards requiring a structured communication process focused on individual patient care [29].

Our findings also suggest that predictable resident and attending schedules, and a smaller number of attendings, may facilitate multiple types of continuity, including resident- and team attending-level longitudinal continuity and continuity of supervision, for same-day and routine visits. These findings are consistent with evidence that block models increase longitudinal continuity with resident providers [30], and with Gupta et al.’s “Clinic First Model.” This model, based on qualitative findings from a multi-site study, recommends eliminating tensions between inpatient and outpatient duties and developing a small core of attendings to increase longitudinal continuity, improve resident training, and decrease provider burnout [31]. Dedicated outpatient time may also spur more physicians to choose to go into primary care [32]. Notably, 6 of 19 participating sites had transitioned away from the traditional residency model, with 8 more planning to or considering doing so.

Finally, we found that there was recognition of patient care and teaching mission alignment in the context of PCMH, and efforts to adapt care delivery to accommodate both. Achieving this alignment is powerful, but not easy, from both care delivery and training perspectives [25]. However, provision of all types of continuity is both a patient care and educational goal, and to attain these goals, sites used strategies such as having attending physicians and their residents in clinic at the same time to improve continuity of supervision, pairing residents with the same patient panel on opposite ambulatory and inpatient blocks to promote management and informational continuity, and having team-based NP trainees participate in providing coverage for same-day access. This last strategy has been reported elsewhere [8], and is consistent with recommendations for educational curricula that emphasize “strengthening the union between inter-professional learning, team-based practice, and high-value care [20].” From a practice standpoint, however, further development of these strategies may require partnerships between not only physician and nurse educators, but also clinic administrators, front-line care providers, quality improvement professionals, and patients.

In addition to highlighting the variety of strategies for promoting access and continuity in the academic primary care clinic training environment, our findings may also provide a basis for constructing a taxonomy of approaches to support future evaluation. Given these variable approaches, further evaluation is needed to determine, which strategies improve different types of continuity in specific contexts and why, and how these models affect patient outcomes. Because context is so important, context-sensitive evaluation approaches such as realistic and developmental evaluation [33, 34], would be promising, as would the use of mixed methods techniques to capture context, mechanisms, experiences, and outcomes. Further, any evaluation of clinic models designed to promote continuity in academic settings should include evaluation of patients’ experience and perceptions of continuity, including continuity with a care team. Questions to be addressed include: How do patients experience team based continuity, i.e., continuity with more than one professional? What is important to patients concerning continuity, and how does that compare in the context of continuity with a single PCP vs. multiple PCPs vs. a care team that includes nurses and other professionals? How do patients weigh the trade-offs between access and continuity in a range of circumstances (e.g., acute vs. chronic illness) [14]?

This work has limitations. First, we interviewed only one participant at each site, and were not able to conduct site visits that would have provided observation data. Additional interviewees and observations would have provided a more holistic understanding of each site and strengthened validity [21]. Finally, we conducted this work within a publically-funded integrated health system; contextual factors, particularly the presence of electronic health records, variation in reimbursement models, and degree of control of resident schedules, may affect transferability of these findings to other systems and independent practices.

## Conclusions

Same-day access and continuity provided through team-based care are critical PCMH requirements. Academic primary care clinics face challenges in meeting these requirements while trying to ensure a robust training environment for new clinicians. As PCMH becomes a prevalent model of primary care delivery, clinics will need to overcome barriers and implement strategies that fulfill both patient care and teaching missions. In contending with part-time providers and residents with complex schedules, clinics themselves have by necessity re-conceptualized continuity within a team-based model by creating alternatives to same-day visits with a usual provider, coupled with communication strategies. Understanding the effect of these alternative strategies on different types of continuity as well as on patient experience and outcomes are key next steps in the further development and dissemination of effective models for improving continuity and the transition to team-based care in the academic clinic setting.

## Additional file

Additional file 1. Interview Guide.docx. Contains interview guide used during data collection. (DOCX 21 kb)

## Abbreviations

ACGME: Accreditation Council for Graduate Medical Education
NCQA: National Committee for Qualitative Assurance
NP: Nurse Practitioner
PA: Physician Assistant
PACT: Patient Aligned Care Teams
PCMH: Patient-centered medical home
PCP: Primary care physician
RN: Registered Nurse
VHA: Veterans Health Administration
VISN: Veterans Integrated Service Network
