# Supplementary material for: Striving toward team-based continuity: provision of same-day access and continuity in academic primary care clinics
Source: BMC Health Serv Res. 2019 Mar 4;19:145. doi: 10.1186/s12913-019-3943-2 (PMC6399842; doi:10.1186/s12913-019-3943-2)
Supplement: Supplementary file 1 — Interview Guide.docx. Contains interview guide used during data collection. (DOCX 21 kb) [file 12913_2019_3943_MOESM1_ESM.docx]

**Additional File 1: Interview Guide**

**Clinic Structure**

- Total number of attendings (or PCPs for those who don’t work with residents):
  - Full-time in clinic
  - Part-time in clinic – # of half days (or, how many are less than 5 half days of clinic/week), what else do they do?
  - Any NPs or PAs?
- Primary Care team(let) structure:
  - Do you have a team/firm structure? If so, how many do you have and how many teamlets are on each team/firm?
  - How are part-time PCPs and residents distributed across your team(let)s?
    - E.g., separate resident teamlet vs. residents distributed across all teamlets?
  - How many providers does each RNCM support? What type (i.e., full-time PCP, part-time PCP, resident)? How many provider FTEE does each RNCM support?
  - How many residents and attendings are there for each ½ day clinic session? Does it vary across sessions? (getting at teamlet staff daily workload)
    - How many RNs are in clinic each day?
    - How many LPNs are in clinic each day?
  - Are team(let)s with part-time PCPs and/or residents structured differently from those without part-time PCPs and/or residents? If so, how? (e.g., team(let)s with more PCPs has a support staff ratio of 3.2:1 rather than 3:1)
  - How is continuity provided to patients?
    - E.g., Team(let) member (e.g., an RNCM) who provides continuity to patients on each team(let)?

**Resident Model**

- How many residents do you have?
- What is your resident clinical model (e.g., block model [3+1, 4+1, 8+8, 12+12], traditional [e.g., one ½ day of clinic per week, two ½ days of clinic per week])?
- Do individual residents have their own panel of patients, or do a group of residents care for a panel of patients?
- If they share a panel, how do they work together to provide care?
- How big are resident panels?
- Who is the site director for your VA Primary Care residency clinic? What are their other VA roles, if any?

**Coverage for Residents Absent from Clinic:** The following questions refer to patient care when the resident is not in clinic, including response to phone messages and secure messages.

- If an individual resident cares for her own patient panel, who sees/manages her patients when she is absent?
- If a patient needs to be physically seen in clinic and the resident isn’t in clinic, who sees the patient for urgent needs? (i.e., ER, urgent care, staff providers, other residents on PACT team(let), any other resident, other)
  - Does this vary for acute care vs. chronic follow-up?
- What are your expectations of residents when they are not in clinic? Specifically:
  - Do you expect them to follow up on labs, imaging, etc. between face-to-face encounters?
    - If yes, what is the mechanism for doing that? (e.g., automated letters, RNCM follow-up, care management tool)
  - Who manages the resident telephone messages (or secure messages or physical mailbox) when the resident is not in clinic?

**Part-time PCPs**

- What responsibilities do your part-time PCPs have in the Primary Care clinic? E.g., linked to residents/precepting residents, have own panel of patients, float coverage
- How often are part-time PCPs on site when not in clinic?
  - Are PCPs who are not in clinic all of the time available 8:00-4:30 when not in clinic for urgent patient care needs?

**Main Challenges**

- What are the most important barriers to meeting PACT metrics?
- If you could change PACT metrics to accommodate the way care is provided in academic medical centers, what would they look like? Why?
- What are the most important issues or challenges you face in implementing PACT in your academic medical center?
- In your opinion, where does having multiple part-time PCPs and residents on your team create the most challenges?
  - E.g., Communication, ability to meet, provision of face-to-face patient care, provision of patient care between visits, medication refills, patients’ ability to get an appointment in clinic when they need it (access), patients’ ability to set up appointment with their own provider (continuity)
